# Suggestive Diagnostic Process in a Case of Multiple Myeloma with Gastrointestinal Immunoglobulin Light-Chain Amyloidosis Accompanied by Protein-Losing Enteropathy

**DOI:** 10.1155/2021/5533993

**Published:** 2021-05-27

**Authors:** Katsuya Endo, Takehito Ito, Jun Nomura, Keigo Murakami, Shiho Kondo, Tomonori Satoh, Daisuke Fukushi, Yuki Yoshino, Yoshiteru Sasaki, Atsuko Takasu, Takayuki Kogure, Morihisa Hirota, Takayoshi Meguro, Kazuhiro Murakami, Junichi Kameoka, Kennichi Satoh

**Affiliations:** ^1^Division of Gastroenterology, Tohoku Medical and Pharmaceutical University School of Medicine, Sendai, Miyagi, Japan; ^2^Division of Hematology, Tohoku Medical and Pharmaceutical University School of Medicine, Sendai, Miyagi, Japan; ^3^Division of Pathology, Tohoku Medical and Pharmaceutical University School of Medicine, Sendai, Miyagi, Japan

## Abstract

Multiple myeloma is a type of plasma cell neoplasm that produces monoclonal immunoglobulin. Multiple myeloma is known to cause immunoglobulin light-chain (AL) amyloidosis, which frequently involves the kidney and heart. Bone pain or fractures caused by osteolytic lesions and physical disorders related to renal or cardiac AL amyloidosis are major initial symptoms in multiple myeloma. Multiple myeloma diagnosed from the gastrointestinal symptoms is rare. We report a case of an 80-year-old man with multiple myeloma accompanied by gastrointestinal AL amyloidosis and secondary protein-losing enteropathy. The diagnostic process was suggestive, in that diarrhea and refractory leg edema related to protein-losing enteropathy were the primary symptoms and the trigger for making a sequential diagnosis of gastrointestinal AL amyloidosis and underlying multiple myeloma. This case is highly suggestive, in that multiple myeloma with gastrointestinal AL amyloidosis should be considered one of the background diseases of protein-losing enteropathy.

## 1. Introduction

Multiple myeloma (MM) is a type of plasma cell neoplasm that produces monoclonal immunoglobulin. MM is known to be associated with immunoglobulin light-chain (AL) amyloidosis, which is characterized by the extracellular deposition of fibrils composed of monoclonal light chains. About 12%–15% of patients with MM will be found to have AL amyloidosis [1]. The kidney and heart are the most frequently involved organs in AL amyloidosis, whereas the gastrointestinal tract is rarely involved [2, 3]. It is difficult to diagnose MM in the early stage because the clinical symptoms may be few or none at the initial phase of the disease. As the disease progresses, various systemic symptoms or signs appear that can be the trigger for diagnosing MM. The initial clinical symptoms or signs of MM are mainly related to the infiltration of neoplastic plasma cells into the bone or to concomitant renal or cardiac AL amyloidosis [2]. MM diagnosed from the symptoms due to gastrointestinal AL amyloidosis is rare.

We experienced a case of MM with gastrointestinal AL amyloidosis accompanied by secondary protein-losing enteropathy (PLE). Initial symptoms related to PLE contributed to the sequential diagnosis of gastrointestinal AL amyloidosis and underlying MM. The case indicates that MM with gastrointestinal AL amyloidosis should be considered one of the background diseases of PLE. Here, we present the case, focusing on its highly suggestive diagnostic process.

## 2. Case Presentation

**Figure 1:**
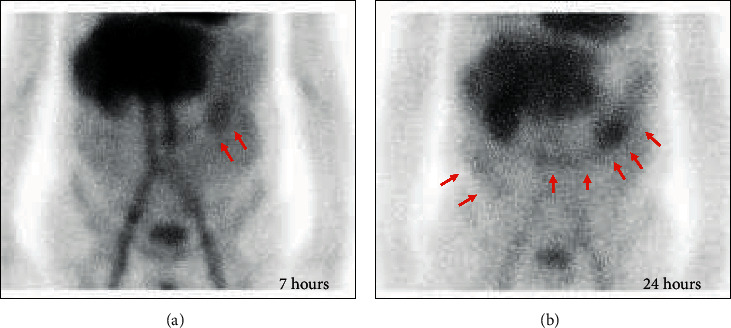
Scanning images of 99 mTc-labeled human serum albumin (HSA) scintigraphy. 99 mTc-labeled HSA scintigraphy revealed albumin leakage from the gastrointestinal tract (arrows). (a) At 7 hours of scanning, collection of the radiotracer was found in the left upper portion of the abdomen, indicating albumin loss into the small intestine. (b) At 24 hours of scanning, diffuse collection of the radiotracer was found in the right and upper portions of the abdomen, indicating albumin loss into the colon.

An 80-year-old man was admitted to our hospital with a 2-week history of severe lower extremity edema, diarrhea, and shortness of breath. He had a prior history of hypertension and diabetes mellitus. On admission, the patient's vital signs were within normal limits. Physical examination revealed severe pitting edema on the bilateral lower legs. Laboratory studies found anemia (hemoglobin 11.2 g/dL), hypoproteinemia (total protein 4.9 g/dL), hypoalbuminemia (3.0 g/dL), elevated serum creatinine (1.27 mg/dL), hypercalcemia (corrected calcium 11.5 mg/dL), decrease in serum immunoglobulin, and urinary protein of 1.3 g per day (Table 1). No significant pathogen was found in the stool culture test. Chest X-ray and chest computed tomography (CT) revealed moderate bilateral pleural effusion. Electrocardiography (ECG) showed low QRS voltage. Echocardiography showed mild tricuspid regurgitation and an ejection fraction of 56%. The B-type natriuretic peptide (BNP) level was 511.6 pg/mL, and the cardiac troponin T (TnT) level was 0.101 ng/mL, suggesting increased cardiac load. We immediately started primary treatment with diuretics; however, both his pleural effusions and his leg edema were extremely refractory. Because persistent hypoproteinemia and chronic diarrhea were observed, PLE was considered as a possible underlying disorder. ^99 m^Tc-labeled human serum albumin (HSA) scintigraphy revealed leakage of albumin from the gastrointestinal tract (Figure 1).

**Figure 2:**
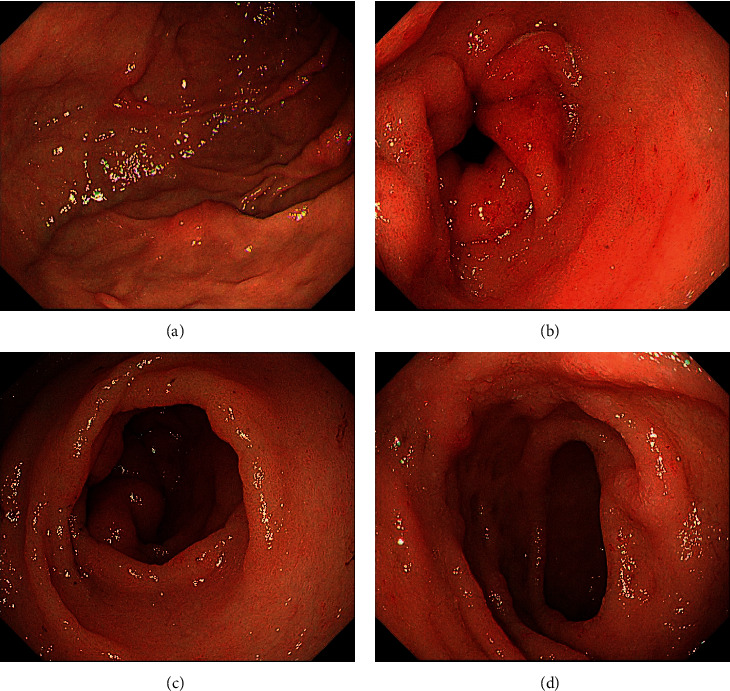
Findings of esophagogastroduodenoscopy (EGD). EGD revealed some elevated lesions in the gastric fornix (a) and moderate edema with redness in the gastric antrum and duodenum (b–d).

**Figure 3:**
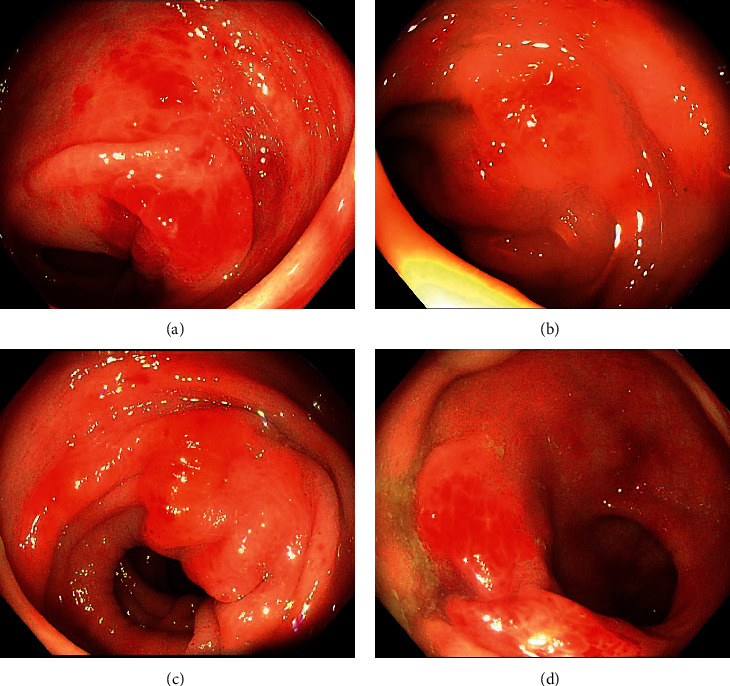
Findings of colonoscopy: (a) descending colon, (b and c) sigmoid colon, (d) rectum. Colonoscopy showed erythema and moderate edema in the entire colon and multiple submucosal tumor-like wall thickenings in the left-sided colon and the rectum.

**Figure 4:**
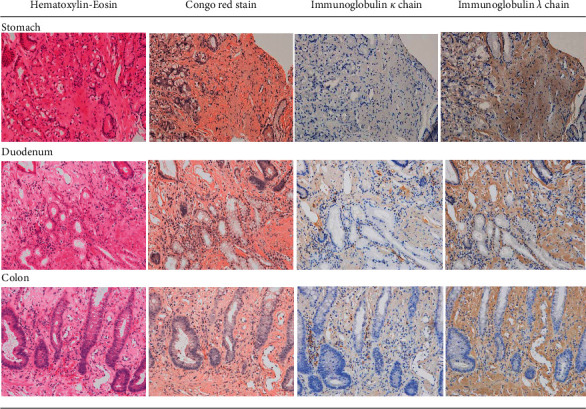
Histopathological findings of gastrointestinal biopsy. Histopathological examination showed Congo-red-positive amyloid deposits in the muscularis mucosae and submucosa of the stomach, duodenum, colon, and rectum. Immunohistostaining showed that the amyloid fibrils were composed of immunoglobulin *λ* light chains.

We diagnosed the patient with PLE. Following the diagnosis of PLE, we examined the gastrointestinal tract by esophagogastroduodenoscopy (EGD) and total colonoscopy (TCS). EGD revealed some elevated lesions in the gastric fornix and moderate edema with redness in the gastric antrum and duodenum (Figure 2). TCS showed erythema and moderate mucosal edema in the entire colon and multiple submucosal tumor-like wall thickenings in the left-sided colon and the rectum (Figure 3). Histopathological examination showed Congo-red-positive amyloid deposits in the muscularis mucosae and submucosa of the stomach, duodenum, colon, and rectum. Immunohistostaining showed that the amyloid fibrils were composed of immunoglobulin *λ* light chains (Figure 4). The amyloid fibril deposits were also confirmed by detecting green birefringence via a polarized light microscope.

**Figure 5:**
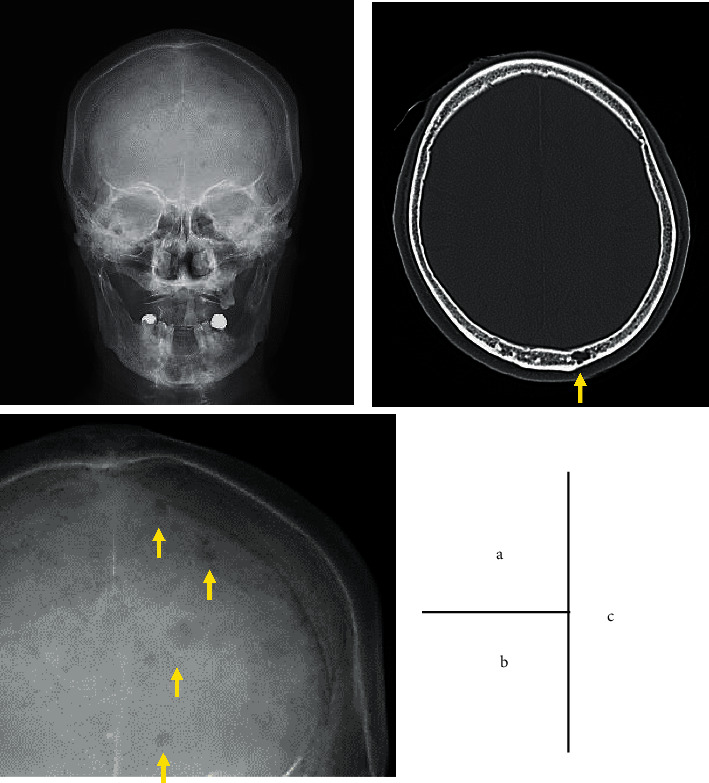
Osteolytic lesions of the skull. Head skeletal X-rays (a and b) and computed tomography (CT) (c) showed multiple, lytic, and punched-out lesions in the skull (arrows).

**Figure 6:**
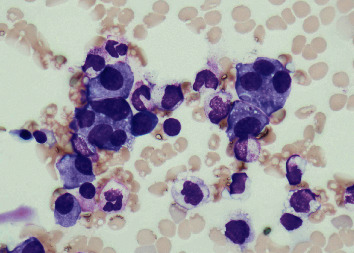
Findings of bone marrow aspiration. Bone marrow aspiration demonstrated an increase of atypical plasma cells of up to 28.8% of the marrow elements.

Based on these findings, the patient was diagnosed with gastrointestinal AL amyloidosis. The diagnosis of gastrointestinal AL amyloidosis strongly suggested that the patient might have MM in the background. Subsequent urinary protein immunoelectrophoresis revealed that *λ*-type Bence Jones protein was positive. An additional head skeletal X-ray showed punched-out lesions, and head CT showed multiple osteolytic lesions in the skull (Figure 5). Bone marrow aspiration showed an increase of atypical plasma cells of up to 28.8% of the marrow elements (Figure 6). Flow cytometry gating on CD38 for bone marrow elements revealed that CD19^–^CD56^+^ cells accounted for 92.5%. Based on these findings, the patient was finally diagnosed with MM with gastrointestinal AL amyloidosis [4]. The patient's PLE was considered a secondary disorder caused by gastrointestinal AL amyloidosis.

The patient was treated with bortezomib and dexamethasone immediately after the diagnosis. However, his general condition deteriorated rapidly, and he died after 7 days of chemotherapy. The direct cause of the patient's early death was renal failure due to the rapid progression of MM.

## 3. Discussion

AL amyloidosis is a major systemic complication in MM. The most frequently involved organs in AL amyloidosis are the kidney and heart; gastrointestinal AL amyloidosis is a rare complication in MM [2]. The present case is a case of MM with gastrointestinal AL amyloidosis accompanied by secondary PLE. Initial symptoms related to PLE led to the sequential diagnosis of gastrointestinal AL amyloidosis and underlying MM. Our case prompts a significant discussion on the process of diagnosis of MM.

In patients with MM, various symptoms or signs can be found as the disease progresses. A previous study of 1027 patients with MM reported that anemia was the most frequent clinical feature (73%), followed by bone pain (58%), elevated serum creatinine (48%), fatigue (32%), hypercalcemia (28%), and weight loss (24%) [5]. Among the most characteristic initial clinical symptoms is bone pain or pathologic fracture caused by osteolytic lesions. The frequency of bone pain in patients with MM has been reported to be approximately 60% [5]. Osteolytic lesions, which are frequently found in the skull, can be identified by plain skeletal radiography or cross-sectional imaging [6]. Hypercalcemia resulting from osteolysis is also an important finding in blood chemistry tests. Our patient did not complain of bone pain, although the laboratory tests showed hypercalcemia at admission. Retrospectively, the patient's hypercalcemia could be regarded as a typical finding of osteolysis due to MM progression; however, it was difficult to directly observe underlying MM at admission. In our patient, typical osteolytic lesions in the skull were confirmed by plain skull X-ray and CT, which were performed after the diagnosis of gastrointestinal AL amyloidosis. Generally, clinical signs and symptoms of MM other than bone lesions include general fatigue and body weight loss. However, these are nonspecific to MM and are common in other diseases.

Clinical symptoms or signs related to concomitant AL amyloidosis can also be the trigger for diagnosing MM [7]. Renal dysfunction, asymptomatic proteinuria, or clinically apparent nephrotic syndrome due to renal AL amyloidosis may trigger the diagnosis of MM. Unexplained heart failure and arrhythmia due to cardiac involvement often lead to the identification of underlying MM. In the present case, renal dysfunction, proteinuria, low voltage in the ECG, and elevated levels of both BNP and TnT were found; hence, we retrospectively speculated that the patient had renal and cardiac AL amyloidosis with a high probability. However, it was difficult for us to directly observe underlying systemic AL amyloidosis and MM based only on the renal and cardiac findings in the actual clinical setting at admission. Instead, we were able to reach the diagnosis of MM by identifying PLE and underlying gastrointestinal AL amyloidosis. Immediately after identifying PLE and gastrointestinal amyloidosis, the final diagnosis of MM was made based on the international diagnosis criteria [4]. This case is rare and suggestive in that gastrointestinal amyloidosis, rather than cardiac or renal amyloidosis, triggered the discovery of MM.

**Figure 7:**
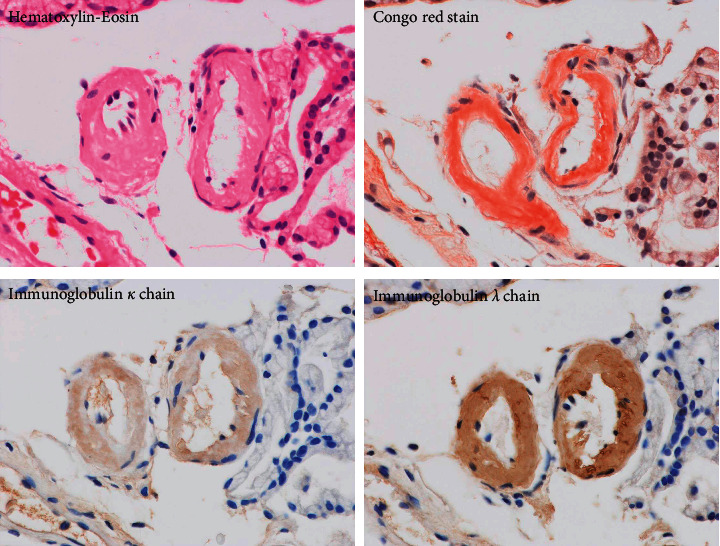
Amyloid deposits in the vascular wall. Amyloid deposits were found in the submucosal vascular walls of the duodenum. (a) Hematoxylin eosin. (b) Congo red stain. (c) Immunoglobulin *κ* chain. (d) Immunoglobulin *λ* chain.

Gastrointestinal AL amyloidosis is one of the rare complications of MM. Patients with gastrointestinal AL amyloidosis were reported to have more organ involvement, more advanced disease, and worse prognosis than those without gastrointestinal involvement [8]. Gastrointestinal AL amyloidosis has various clinical manifestations, including abdominal pain, nausea, diarrhea, malabsorption, gastrointestinal bleeding, and PLE [9–12]. PLE is a quite rare but severe complication of gastrointestinal amyloidosis in an advanced stage. Although the precise pathogenesis of PLE accompanied by gastrointestinal AL amyloidosis has not been clarified, autonomic neuropathy and/or amyloid deposits in vessels and lymphatics are speculated to cause excessive loss of serum protein to the gastrointestinal tract [12]. In the present case, amyloid deposition was found in submucosal vascular walls of the duodenum (Figure 7), which could be one of the major causes of PLE. At admission, this patient presented with PLE-related clinical symptoms, such as refractory peripheral edema, chronic diarrhea, hypoproteinemia, and hypoalbuminemia. These initial symptoms prompted us to perform 99 mTc HSA scintigraphy and led to the diagnosis of PLE. Endoscopic examination and biopsy for investigating the cause of PLE contributed to the successful diagnosis of gastrointestinal AL amyloidosis. The endoscopic findings in gastrointestinal amyloidosis, such as erythema, granular appearance, erosions and ulcers, friability, polypoid protrusions, and wall thickening, have been reported to be nonspecific [10, 13–16]. The endoscopic features of the present patient also had several unusual findings, such as elevated lesions, edema, erythema, and submucosal tumor-like wall thickening, which are uncommon in other diseases, such as infectious colitis or inflammatory bowel disease. The histopathological findings in our patient were compatible with gastrointestinal AL amyloidosis. The Congo-red-positive deposits in the mucosa and submucosa were identified as immunoglobulin *λ* light chains by immunohistochemical staining. The diagnosis of gastrointestinal AL amyloidosis prompted us to identify *λ*-type Bence Jones protein in the urine, osteolytic lesions, and the increase of atypical plasma cells in the bone marrow, which led to the final diagnosis of MM. The sequential diagnostic process in our case can be highly suggestive, in that MM with gastrointestinal AL amyloidosis should be considered one of the background diseases of PLE.

In conclusion, we experienced the unique and suggestive diagnostic process in a case of MM with gastrointestinal AL amyloidosis accompanied by PLE. The present case suggests that the presence of PLE can lead to a diagnosis of gastrointestinal AL amyloidosis and its underlying MM.

## Figures and Tables

**Table 1:** Laboratory data on admission.

WBC	5500	/*µ*L	AST	20	IU/L	TP	4.9	g/dL
Neut	69.0	%	ALT	14	IU/L	Alｂ	3.0	g/dL
Eosi	0.6	%	LDH	167	IU/L	T-chol	130	mg/dL
Baso	0.4	%	T-bil	0.8	mg/dL	TG	110	mg/dL
Lymph	21.4	%	D-bil	0.1	mg/dL	HDL-C	36	mg/dL
Mono	8.6	%	ALP	306	U/L	FBS	103	mg/dL
RBC	319	×104/*µ*L	gGTP	56	IU/L	HbA1c	6.0	%
Hb	11.2	g/dL	BUN	52	mg/dL	CRP	0.32	mg/dL
Ht	33.5	%	Cr	1.27	mg/dL	IgG	635	mg/dL
MCV	105	fL	Na	137	mEq/L	IgA	104	mg/dL
MCH	35	Pg	K	4.5	mEq/L	IgM	19	mg/dL
MCHC	33.3	%	Cl	100	mEq/L			
Plt	19.1	×104/*µ*L	Ca	10.6	mg/dL			
			Ca (corrected for Alb level)	11.5	mg/dL	Urinary protein	1.3	g/day

## Data Availability

The data used to support the findings of this study are available from the corresponding author upon request.
